# A Case of Annular Pustular Psoriasis Possibly Triggered by Cabozantinib and Edoxaban During Nivolumab Therapy

**DOI:** 10.1111/1346-8138.70301

**Published:** 2026-05-03

**Authors:** Mana Nagatake, Hiroto Horikawa, Risa Kakuta, Takeya Adachi, Keishiro Fukumoto, Masayuki Amagai, Yoshihiro Ito

**Affiliations:** ^1^ Department of Dermatology Keio University School of Medicine Tokyo Japan; ^2^ Allergy Center Keio University Hospital Tokyo Japan; ^3^ Department of Urology Keio University School of Medicine Tokyo Japan

Dear Editor,

1

Annular pustular psoriasis (APP) is a rare variant of psoriasis that occasionally presents as a drug eruption. Here, we report a case of APP possibly triggered by cabozantinib and edoxaban during nivolumab administration.

A 73‐year‐old man received combination therapy with nivolumab and cabozantinib for renal cell carcinoma. Four weeks later, progressive erythematous eruptions that were unresponsive to topical corticosteroids appeared on the trunk. Physical examination revealed annular erythema with peripheral scaling and occasional pustules on the trunk and extremities without systemic manifestations (Figure 1a,b). Bacterial cultures of the pustule and potassium hydroxide (KOH) preparations for fungal elements were negative. Histopathological examination revealed mild epidermal hyperplasia, subcorneal neutrophilic microabscesses, adjacent epidermal infiltration, granular layer loss, focal vacuolar changes at the dermo‐epidermal junction, and superficial perivascular lymphocytic infiltration (Figure 1c,d), consistent with APP.

**FIGURE 1:**
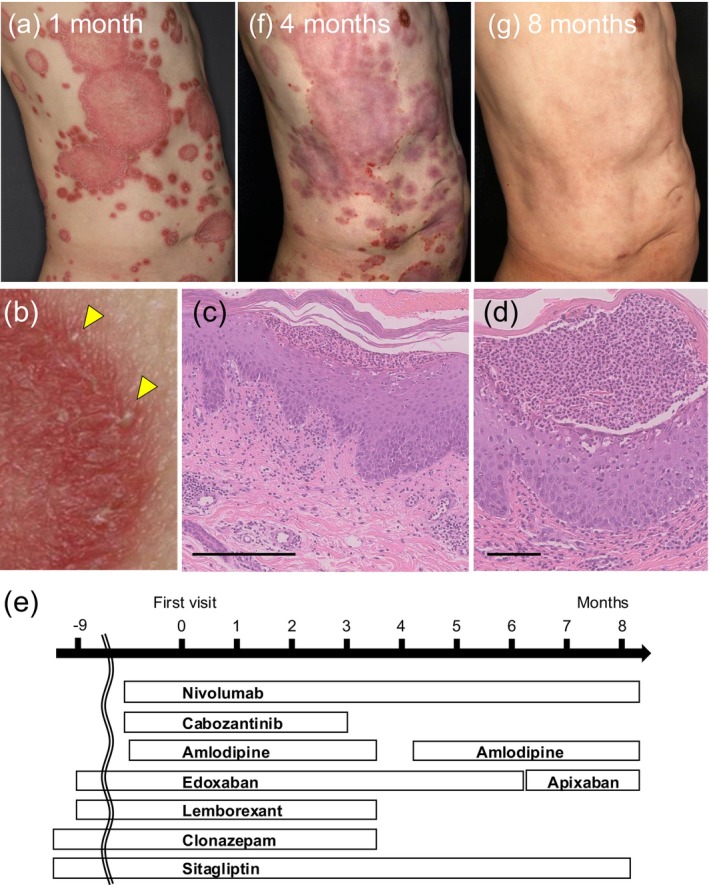
Clinical presentation and drug administration timeline. (a) Clinical appearance at 1 month, showing annular erythematous plaques with occasional pustules on the trunk. (b) A magnified view of the lesion, highlighting small pustules (yellow arrowheads) distributed along the erythematous rim. (c) Histopathology showing mild epidermal hyperplasia, subcorneal neutrophilic microabscesses, and adjacent epidermal infiltration, granular layer loss, focal vacuolar changes at the dermoepidermal junction, and superficial lymphocytic infiltration. Scale bar = 200 μm. (d) High‐power view showing neutrophil infiltration of the subcorneal space and within the epidermis. Scale bar = 100 μm. (e) Timeline of administered medications. (f) Partial improvement of the lesions observed at 1 month after discontinuation of cabozantinib, although new eruptions continued. (g) Complete resolution of the lesions at 8 months after discontinuation of edoxaban.

After suspecting a drug‐induced etiology, the patient's medication history was reviewed, and among seven medications, nivolumab, cabozantinib, and amlodipine had been newly introduced (Figure 1e). Lymphocyte transformation tests (LTTs) were performed for newly initiated drugs, excluding nivolumab. Cabozantinib yielded a positive result (stimulation index [SI] 3.6; cutoff > 1.8), whereas amlodipine yielded a negative result (SI 1.2). Cabozantinib discontinuation led to partial improvement; however, new lesions continued to appear (Figure 1f). Given the incomplete resolution, preexisting medications were temporarily withdrawn; however, cessation of amlodipine, lemborexant, and clonazepam had no clinical effect. The LTT for edoxaban was positive (SI 2.1), and replacement with apixaban resulted in complete resolution of skin lesions (Figure 1g). These findings supported the diagnosis of drug‐induced APP, likely triggered by cabozantinib and edoxaban. Notably, nivolumab administration was continued for 2 years without adverse skin events, while maintaining cancer control.

APP can be induced by various factors, including medications, infections, and hormonal fluctuations [1]. Among these medications, oral agents such as Angiotensin‐Converting Enzyme (ACE) inhibitors, beta‐blockers, corticosteroids, and over‐the‐counter cold medications have been reported as potential causes [2, 3, 4]. During immune checkpoint inhibitor (ICI) therapy, T cell activation induced by agents such as nivolumab may lead to immune dysregulation, reducing the threshold for drug‐induced hypersensitivity reactions that would otherwise be well tolerated [5]. In the present case, no symptoms were observed during edoxaban monotherapy; however, cutaneous manifestations emerged after nivolumab initiation. Nivolumab could be continued without the recurrence of skin symptoms once the suspected drugs were discontinued, suggesting that nivolumab may have contributed to an aberrant immune milieu that indirectly triggered APP in response to multiple agents.

To our knowledge, there are no previous reports of APP caused by two concomitant drugs during ICI therapy. Both cabozantinib and edoxaban were suspected as causative agents, based primarily on the close temporal relationship between drug discontinuation and rapid clinical improvement, with LTT providing supportive evidence.

We presented the first case of APP likely triggered by two concomitant drugs during ICI therapy. A careful review of concomitant medications, with close attention to the clinical course following drug discontinuation, was instrumental in identifying causative agents. Importantly, this approach enabled the safe continuation of ICI therapy without the recurrence of dermatological adverse events.

## Funding

The authors have nothing to report.

## Ethics Statement

Approval of the research protocol by an Institutional Review Board: N/A.

Informed Consent: The patient's consent was obtained for the publication of his photographs and medical information, both in print and online.

Registry and Registration No. of study/trial: N/A.

Animal studies: N/A.

## Conflicts of Interest

The authors declare no conflicts of interest.

## Data Availability

Research data are not shared.
